# Anti-Mullerian hormone attenuates both cyclophosphamide-induced damage and PI3K signalling activation, while rapamycin attenuates only PI3K signalling activation, in human ovarian cortex *in vitro*

**DOI:** 10.1093/humrep/dead255

**Published:** 2023-12-09

**Authors:** Roseanne Rosario, Hazel L Stewart, Norah Spears, Evelyn E Telfer, Richard A Anderson

**Affiliations:** Biomedical Sciences, University of Edinburgh, Edinburgh, UK; Centre for Reproductive Health, Institute for Regeneration and Repair, University of Edinburgh, Edinburgh, UK; Centre for Reproductive Health, Institute for Regeneration and Repair, University of Edinburgh, Edinburgh, UK; Biomedical Sciences, University of Edinburgh, Edinburgh, UK; Biomedical Sciences, University of Edinburgh, Edinburgh, UK; Institute of Cell Biology, University of Edinburgh, Edinburgh, UK; Centre for Reproductive Health, Institute for Regeneration and Repair, University of Edinburgh, Edinburgh, UK

**Keywords:** chemotherapy effects, cyclophosphamide, chemoprotection, anti-Mullerian hormone, human ovary, fertility preservation

## Abstract

**STUDY QUESTION:**

What are the effects of cyclophosphamide exposure on the human ovary and can anti-Mullerian hormone (AMH) and rapamycin protect against these?

**SUMMARY ANSWER:**

Exposure to cyclophosphamide compromises the health of primordial and transitional follicles in the human ovarian cortex and upregulates PI3K signalling, indicating both direct damage and increased follicular activation; AMH attenuates both of these chemotherapy-induced effects, while rapamycin attenuates only PI3K signalling upregulation.

**WHAT IS KNOWN ALREADY:**

Studies primarily in rodents demonstrate that cyclophosphamide causes direct damage to primordial follicles or that the primordial follicle pool is depleted primarily through excessive initiation of follicle growth. This increased follicular activation is mediated via upregulated PI3K signalling and/or reduced local levels of AMH production due to lost growing follicles. Furthermore, while rodent data show promise regarding the potential benefits of inhibitors/protectants alongside chemotherapy treatment to preserve female fertility, there is no information about the potential for this in humans.

**STUDY DESIGN, SIZE, DURATION:**

Fresh ovarian cortical biopsies were obtained from 17 healthy women aged 21–41 years (mean ± SD: 31.8 ± 4.9 years) at elective caesarean section. Biopsies were cut into small fragments and cultured for 24 h with either vehicle alone (DMSO), the active cyclophosphamide metabolite 4-hydroperoxycyclophosphamide (4-HC) alone, 4-HC + rapamycin or 4-HC+AMH. Two doses of 4-HC were investigated, 0.2 and 2 μM in separate experiments, using biopsies from seven women (aged 27–41) and six women (aged 21–34), respectively. Biopsies from four women (aged 28–38) were used to investigate the effect of rapamycin or AMH only.

**PARTICIPANTS/MATERIALS, SETTING, METHODS:**

Histological analysis of ovarian tissue was undertaken for follicle staging and health assessment. Western blotting and immunostaining were used to assess activation of PI3K signalling by measuring phosphorylation of AKT and phosphorylated FOXO3A staining intensity, respectively.

**MAIN RESULTS AND THE ROLE OF CHANCE:**

Exposure to either dose of 4-HC caused an increase in the proportion of unhealthy primordial (*P* < 0.0001, both doses) and transitional follicles (*P* < 0.01 for low dose and *P* < 0.01 for high dose) compared to vehicle. AMH significantly reduced follicle damage by approximately half in both of the investigated doses of 4-HC (*P* < 0.0001), while rapamycin had no protective effect on the health of the follicles. Culture with AMH or rapamycin alone had no effect on follicle health. Activation of PI3K signalling following 4-HC exposure was demonstrated by both Western blotting data showing that 4-HC increased in AKT phosphorylation and immunostaining showing increased phosphorylated FOXO3A staining of non-growing oocytes. Treatment with rapamycin reduced the activation of PI3K signalling in experiments with low doses of 4-HC while culture with AMH reduced PI3K activation (both AKT phosphorylation and phosphorylated FOXO3A staining intensity) across both doses investigated.

**LIMITATIONS, REASONS FOR CAUTION:**

These *in vitro* studies may not replicate *in vivo* exposures. Furthermore, longer experiment durations are needed to determine whether the effects observed translate into irreparable deficits of ovarian follicles.

**WIDER IMPLICATIONS OF THE FINDINGS:**

These data provide a solid foundation on which to explore the efficacy of AMH in protecting non-growing ovarian follicles from gonadotoxic chemotherapies. Future work will require consideration of the sustained effects of chemotherapy treatment and potential protectants to ensure these agents do not impair the developmental competence of oocytes or lead to the survival of oocytes with accumulated DNA damage, which could have adverse consequences for potential offspring.

**STUDY FUNDING/COMPETING INTEREST(S):**

This work was supported by grants from TENOVUS Scotland, the Academy of Medical Sciences (to R.R.), the Medical Research Council (G1100357 to R.A.A., MR/N022556/1 to the MRC Centre for Reproductive Health), and Merck Serono UK (to R.A.A.). R.R., H.L.S., N.S., and E.E.T. declare no conflicts of interest. R.A.A. reports grants and personal fees from Roche Diagnostics and Ferring Pharmaceuticals, and personal fees from IBSA and Merck outside the submitted work.

**TRIAL REGISTRATION NUMBER:**

N/A.

## Introduction

The effects of chemotherapy treatment on clinical aspects of ovarian function are increasingly well-documented. These include varying levels of damage resulting in reduced fertility and chances of pregnancy through to complete loss of the non-renewable primordial follicles and hence premature ovarian insufficiency (Oktem and Oktay, 2007b; Loren *et al.*, 2013; Anderson *et al.*, 2018). The degree of damage and associated risk of infertility depends on the dose and type of chemotherapeutic agent and on the patient’s age at the time of treatment, with older women being at greater risk. The impact on the quiescent primordial follicles and surrounding stromal environment is central to determining the long-term risk of fertility loss and oestrogen deficiency, as it is this non-renewable reserve of primordial follicles that underpins lifelong female fertility. Importantly, while these reproductive consequences can manifest immediately, they will frequently not do so until many years after chemotherapy treatment has ceased, with the length of this gap being related to the size of the primordial follicle store at the time of treatment, particularly for pre-pubertal girls who have a high ovarian reserve before treatment.

Primordial follicles have four possible fates: to maintain quiescence, to undergo direct cell death, to undergo growth activation but with the developing follicle later dying by atresia, or to activate and grow until fully mature and able to ovulate (McGee and Hsueh, 2000; Ford *et al.*, 2020; Telfer *et al.*, 2023). This delicate balance underpins female reproductive function and, critically, its duration. The evidence describing the principal mechanisms underlying primordial follicle loss following chemotherapy is conflicting. Studies primarily in rodents demonstrate that chemotherapy agents cause direct damage to primordial follicles including DNA damage within oocytes (Gonfloni *et al.*, 2009; Kerr *et al.*, 2012; Nguyen *et al.*, 2018, 2019; Allen *et al.*, 2020; Lopes *et al.*, 2020; Stringer *et al.*, 2020). However, others suggest that specific chemotherapeutics (including the alkylating agents most associated with ovarian toxicity such as cyclophosphamide and cisplatin) cause depletion of the primordial follicle pool primarily through excessive follicle activation. This phenomenon, often referred to as ‘burnout’ (Kalich-Philosoph *et al.*, 2013; Chang *et al.*, 2015; Goldman *et al.*, 2017; Lande *et al.*, 2017; Sonigo *et al.*, 2019; Shai *et al.*, 2021) may be a consequence of upregulated phosphatidylinositol-3 kinase (PI3K) signalling. In the ovary, PI3K signalling is a fundamental intracellular pathway through which diverse local factors converge to govern initial primordial follicle recruitment; this is effected in oocytes via the transcription factor forkhead box 3A (FOXO3A) (John *et al.*, 2008). Excessive follicle activation may also arise as a result of released inhibition from a dwindling population of developing follicles due to increased apoptosis in that follicle population, thus leading to reduced local levels of anti-Mullerian hormone (AMH) production from that developing follicle pool (Durlinger *et al.*, 2002; Sonigo *et al.*, 2019). Investigations into the effects of chemotherapy on human primordial follicles are much more limited, with analysis of *in vivo* chemotherapy-exposed ovarian tissue (Devos *et al.*, 2023) and human ovarian xenograft models supporting apoptotic death of primordial follicle oocytes as the main mechanism for follicle loss (Oktem and Oktay, 2007a; Soleimani *et al.*, 2011), while analysis of *in vivo* alkylating agent-exposed ovarian tissue (Shai *et al.*, 2021) and *in vitro* culture studies provide evidence of excessive follicle activation (Lande *et al.*, 2017). Strikingly, there are also a couple of reports (Bellusci *et al.*, 2019; Kashi *et al.*, 2023) that support the contribution of both these mechanisms of primordial follicle loss following chemotherapy treatment, with both these studies investigating the effects of cyclophosphamide using *in vivo* and *in vitro* rodent models.

The field of fertility preservation has developed rapidly, with improvements in oocyte storage and freezing of ovarian tissue (reviewed in Anderson *et al.* (2015, 2020)); however, these treatments can be complex, invasive, and time consuming. Therefore, there has been significant interest in the development of therapies to help protect the ovary from unwanted damage at the time of cancer treatment (Spears *et al.*, 2019). Rapamycin is a specific inhibitor of mTORC1, a key component in the PI3K signalling pathway, and its pharmacological administration has been shown to prevent global follicular activation and to preserve the otherwise depleted ovarian reserve in *Pten*-deficient mice (Adhikari *et al.*, 2013). Since then, numerous studies have demonstrated the effectiveness of rapamycin in protecting the rodent ovary from both chemotherapy-induced primordial follicle damage and excessive follicle activation. These data have been generated using *in vitro* and *in vivo* models of chemotherapy exposure, investigating the effects of both cyclophosphamide and cisplatin (Zhou *et al.*, 2017; Xie *et al.*, 2020; Chen *et al.*, 2022). Data collected following the use of the mTOR inhibitor everolimus to protect against cisplatin-induced ovarian toxicity further support inhibition of mTOR as a pathway to chemoprotection (Goldman *et al.*, 2017; Tanaka *et al.*, 2018). It is generally accepted that AMH is a paracrine regulator of primordial follicle activation, with exogenous application inhibiting primordial follicle activation in *in vitro* cultures of rodent and bovine ovary tissue (Durlinger *et al.*, 2002; Nilsson *et al.*, 2007; Kano *et al.*, 2017), although human data are more scarce (Carlsson *et al.*, 2006). Regarding chemoprotection, mice that were administered supraphysiological doses of AMH using either an adeno-associated virus serotype 9 (AAV9) gene therapy vector or recombinant protein alongside carboplatin, doxorubicin, or cyclophosphamide had significantly more primordial follicles than did controls (Kano *et al.*, 2017). Furthermore, recombinant AMH prevented cyclophosphamide-induced primordial follicle depletion and rescued fertility in two pre-pubertal mouse models (Roness *et al.*, 2019; Sonigo *et al.*, 2019). Here, the authors also demonstrated that PI3K signalling and FOXO3A phosphorylation were decreased after AMH injection (Sonigo *et al.*, 2019). A recent study investigated the premise of attenuating both the apoptotic and follicular activation effects of cyclophosphamide using combined treatment with temsirolimus (an mTOR inhibitor) and AMH, and a complete protection of the primordial follicle reserve in the treated mice was observed (Kashi *et al.*, 2023). Collectively, these rodent data show that concomitant delivery of potential inhibitors/protectants alongside chemotherapy treatment has promise for preserving female fertility, however, there is no information about the potential for this in humans.

In this work, we have investigated the effects of cyclophosphamide on human primordial follicle damage and primordial follicle activation using an established ovarian cortical tissue culture model (Telfer *et al.*, 2008). Since cyclophosphamide is a pro-drug, *in vitro* experiments typically use the directly damage-inducing metabolites of cyclophosphamide to determine downstream effects. Here we have used the metabolite 4-hydroperoxycyclophosphamide (4-HC), which is a cell-permeable agent that leads to a series of spontaneous reactions (without enzymatic aid), resulting in the production of phosphoramide mustard, which is generally accepted as the reactive alkylating agent of therapeutic consequence (Ludeman, 1999). Alongside culture with 4-HC, we have investigated the effectiveness of rapamycin and AMH to protect human primordial follicles from the negative consequences of cyclophosphamide exposure. Our data show that exposure to 4-HC for 24 h affected the health of primordial and transitional follicles and upregulated PI3K signalling in these non-growing follicles. Furthermore, AMH was able to mitigate the effects of 4-HC on both follicle activation and health whilst addition of rapamycin only attenuated the follicle activation PI3K signalling response. These data therefore identify potential approaches that can be used to ameliorate cyclophosphamide-induced damage in the human ovary.

## Materials and methods

### Ethical approval

Approval of this study to obtain ovarian cortical biopsies after informed consent from women undergoing elective caesarean section was given by the local ethics committee (ref LREC 10/S1101/2).

### Ovarian cortical tissue collection, preparation, and culture

Fresh ovarian cortical biopsies were obtained from 17 healthy women aged 21–41 years (mean ± SD: 31.8 ± 4.9 years) undergoing elective caesarean section; no women had any history of gynaecological pathologies. Ovarian tissue was transported to the laboratory in dissection medium (Leibovitz medium (Invitrogen Ltd, Paisley, UK) supplemented with sodium pyruvate (2 mM), L-glutamine (2 mM) (both Invitrogen Ltd), human serum albumin (HSA) (3 mg/ml), penicillin G (75 μg/ml), and streptomycin (50 μg/ml) (Sigma Chemicals, Poole, Dorset, UK)). In the laboratory, excess stromal and haemorrhagic tissue as well as follicles measuring >80 μm were removed, and the remaining cortical tissue was cut into fragments measuring ∼4 mm long, ∼1 mm wide, and ∼1 mm thick. At least two to three cortical fragments were assigned to one of four treatment groups: vehicle (DMSO), 4-HC alone (Niomech IIT GmbH, Germany, diluted in DMSO), 4-HC+100 nM rapamycin (LC Laboratories, USA, prepared according to manufacturer’s instructions), or 4-HC+200 ng/ml recombinant human AMH (R&D Systems, UK, prepared according to manufacturer’s instructions). Two doses of 4-HC were investigated: a low dose of 0.2 μM and a higher dose of 2 μM. The lower dose corresponds to the lower end of what has been measured in plasma of cancer patients receiving therapeutic doses of cyclophosphamide, and a comparable dose was shown not to affect primordial follicle numbers in a neonatal mouse ovary culture model; the higher dose is comparable to a dose that caused about an ∼80% loss in primordial follicles in mice following a 2-day *in vitro* exposure and subsequent 6-day culture period (Desmeules and Devine, 2006; Luan *et al.*, 2019). Doses similar to 2 μM have been used in other mouse and human ovarian *in vitro* studies; while doses up to 10 μM have been investigated in human, we wanted to avoid non-specific cell death. Note that each dose was investigated in separate experiments. Each fragment was cultured individually in 24-well cell culture plates (Corning B.V. Life Sciences Europe, Amsterdam) in 300 μl of culture medium (McCoy’s 5A medium with bicarbonate with HEPES (20 mM, Invitrogen Ltd), glutamine (3 mM, Invitrogen Ltd), HSA (0.1%), penicillin G (0.1 mg/ml), streptomycin (0.1 mg/ml), transferrin (2.5 μg/ml), selenium (4 ng/ml), human insulin (10 ng/ml) (all obtained from Sigma, UK, unless specified)). Treatments (DMSO vehicle, 4-HC, rapamycin, or AMH) were added to media at the beginning of culture and fragments were cultured for 24 h at 37°C in humidified air with 5% CO_2_. Note, although 4-HC was left in culture media for duration of experiment, the half-life of 4-HC at 37°C is 30 min (Niomech IIT GmbH product datasheet), therefore the chemotherapy is likely to only have an effect during the first few hours of culture, thus mimicking the human exposure period. After culture, the ovarian fragments were snap frozen for Western blotting or fixed in 10% normal buffered formalin for histological and immunological assessment.

### Histological analysis

Fixed ovarian fragments were dehydrated, embedded in paraffin, and serially sectioned at 5 μm thickness. Every 10th section was stained with haematoxylin and eosin, and follicle stage and health were assessed. Follicles were staged according to the following criteria: primordial follicles (oocyte surrounded by flattened granulosa cells), transitional follicles (oocyte surrounded by flattened and at least one cuboidal granulosa cell), primary follicles (oocyte surrounded by one complete layer of cuboidal granulosa cells), or secondary follicles (oocyte surrounded by two or more complete layers of cuboidal granulosa cells). Only primordial and transitional follicles that contained an oocyte nucleus were counted, and when nuclei were more difficult to observe in growing follicles, care was taken to avoid double counting by noting the follicle size and location in each tissue section. In this manuscript, the term non-growing follicles is used to collectively describe primordial and transitional follicles, while growing follicles refers to those at the primary and secondary stages. The health status of follicles was classified according to morphological criteria as previously described (McLaughlin and Telfer, 2010; McLaughlin *et al.*, 2014). Oocytes and granulosa cells were assessed, with unhealthy/degenerating oocytes being eosinophilic or showing shrunken cytoplasm, and/or condensed nuclear chromatin, and/or overall compromised morphology, while granulosa cells were considered unhealthy when the majority of cells within the follicle were irregularly shaped and/or had condensed chromatin. Follicles were classified as unhealthy on the basis of either the oocyte or granulosa cells or a combination of both. Cortical tissue volume was calculated as the sum of the area in mm^2^ of all tissue sections analysed per patient, multiplied by 0.005 (the thickness of each section in mm), multiplied by 10 (since every 10th section was analysed), to give a value in mm^3^; follicle density was then ascertained by dividing the total number of follicles per patient by the volume of tissue analysed and expressing this value as follicle density per mm^3^ (McLaughlin *et al.*, 2015).

### Immunostaining and image analysis

Immunostaining for pFOXO3A was carried out on ovarian cortical sections to interrogate activation of PI3K signalling in primordial and transitional follicle oocytes, according to standard protocols. Incubation with anti-pFOXO3A (ab154786, Abcam, UK) at 1:100 was carried out overnight at 4°C. ImmPRESS^®^ HRP Horse Anti-Rabbit IGG PLUS Polymer Kit (MP-7801, Vector Laboratories, UK) was used for secondary antibody labelling, with subsequent visualization using 3,3′-diaminobenzidine (DAB) (Agilent DAKO, USA). Tissue was incubated with DAB for 10 min, and this was kept consistent across experiments. Slides were counterstained with haematoxylin before mounting and imaged using an Axioscan slide scanner. Image analysis was carried out objectively using FIJI to measure mean grey levels of cytoplasmic pFOXO3A staining (over a scale of 0–256). Oocytes were classified as strong staining with grey levels between 0 and 120, weak staining with grey levels between 121 and 219, and no staining with grey levels between 220 and 256. These data were then processed to calculate the proportion of oocytes with strong, weak, and no pFOXO3A staining.

### Western blotting

Western blotting was used to interrogate activation of PI3K signalling by assessing expression of signalling mediator AKT and its phosphorylated form pAKT. One cortical strip per treatment group was homogenized in RIPA buffer (150 mM NaCl, 1% NP-40, 0.5% sodium deoxycholate, 0.1% sodium dodecyl sulphate, 50 mM Tris–HCl pH8 with freshly added protease and phosphatase inhibitors (Roche, Merck Life Sciences Ltd, UK)). Each sample was then split to allow assessment of AKT and pAKT expression within the same cortical strip. Protein separation was carried out on Mini-PROTEAN TGX precast gels (BioRad, UK) with Laemmli sample buffer. Proteins were transferred onto Immobilon^®^-FL membranes (Millipore, Merck, Ireland). Primary antibodies were incubated overnight at 4°C at the following dilutions: anti-AKT at 1:1000 (cat no. 9272S, Cell Signalling Technology, Netherlands), anti P-AKT at 1:1000 (cat no. 9271S, Cell Signalling Technology), or anti-ACTB (cat no. A1978, Merck). Secondary antibodies were used at 1:10 000 (cat no. A10043, cat no. A32730, ThermoFisher Scientific, UK) and incubated for 1 h at room temperature. Membranes were imaged using a LI-COR Odyssey and analysed with Image Studio Ver 5.2. To analyse, the density of AKT and pAKT, bands were normalized to ACTB density on their respective gels, and the ratio of normalized pAKT densities to normalized AKT densities was then calculated. These values were then used for statistical analyses, with the data plotted relative to the vehicle sample for graphing.

### Statistical analysis

All data are shown as mean ± SEM and were analysed using GraphPad Prism 9 software (GraphPad Software, Inc., San Diego, CA). Mann–Whitney and Fisher’s exact test statistics were carried out as appropriate for Western blotting data and follicle data, respectively. A *P*-value of <0.05 was considered statistically significant.

## Results

### 4-HC exposure affects the health of non-growing follicles in human ovarian cortex *in vitro*, and this is improved by culture with AMH

**Figure 1.**
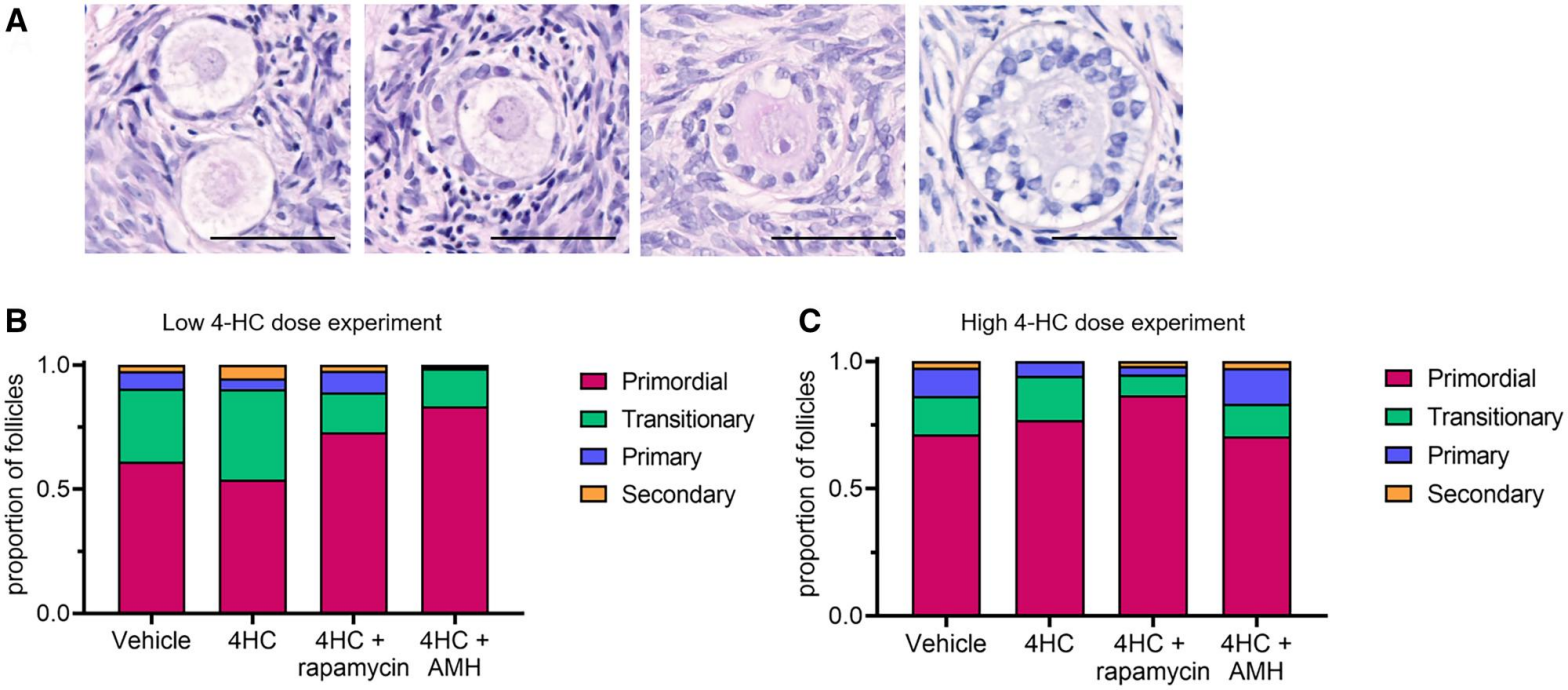
**Staging of follicles in human ovarian cortex following 24 h exposure to the active cyclophosphamide metabolite 4-hydroperoxycyclophosphamide** (**4-HC).** (**A**) Representative histological images of primordial, transitional, primary, and secondary follicles in cultured human ovarian cortical tissue. Scale bar = 50 µm. (**B, C**) Proportions of ovarian follicles classified as primordial, transitional, primary, and secondary follicles in experiments with low 0.2 µM doses of 4-hydroperoxycyclophosphamide (4-HC) (B) and high 2 µM doses of 4-HC (C) (4-HC) with or without the chemoprotectants (anti-Mullerian hormone (AMH) and rapamycin). N = 3–7, 436 follicles counted in total for low-dose experiments. N = 4–6, 1285 follicles counted in total for high-dose experiments.

Human ovarian cortical strips were cultured with 0.2 or 2 µM 4-HC ± rapamycin or AMH for 24 h and histological analyses were undertaken to classify follicle stage and health. A total of 436 follicles from seven patients (aged 27–41) and 1285 follicles from six patients (aged 21–34) were assessed in the low- (0.2 µM) and high-dose (2 µM) 4-HC experiments, respectively (see Supplementary Table S1 for patient age and number of follicles counted). The majority of follicles across both experiments and all treatment groups were non-growing primordial or transitional follicles, with few primary and secondary follicles present after the 24-h culture period (Fig. 1). An analysis of follicle density in the high-dose 4-HC experiments found no significant difference between the four experimental groups (Supplementary Fig. S1). After culture with rapamycin or AMH in combination with 4-HC, the proportion of primordial follicles (73.0 ± 9.4% in the rapamycin+0.2 µM 4-HC group and 83.5 ± 10.8% in the AMH+0.2 µM 4-HC group) appeared higher than in the vehicle group (61.1 ± 9.4%) (Fig. 1B); a similar trend was observed in the rapamycin+2 µM 4-HC versus vehicle data (Fig. 1C). However, it is important to note that these data do not reflect the differences in absolute number of each follicle stage, thus such observations in follicle proportions may be misleading and preclude reliable interpretation regarding effects on follicle survival or growth activation. Therefore, no statistical analyses were undertaken on this staging data, and instead, follicle health data were collected on non-growing and growing follicles separately.

**Figure 2.**
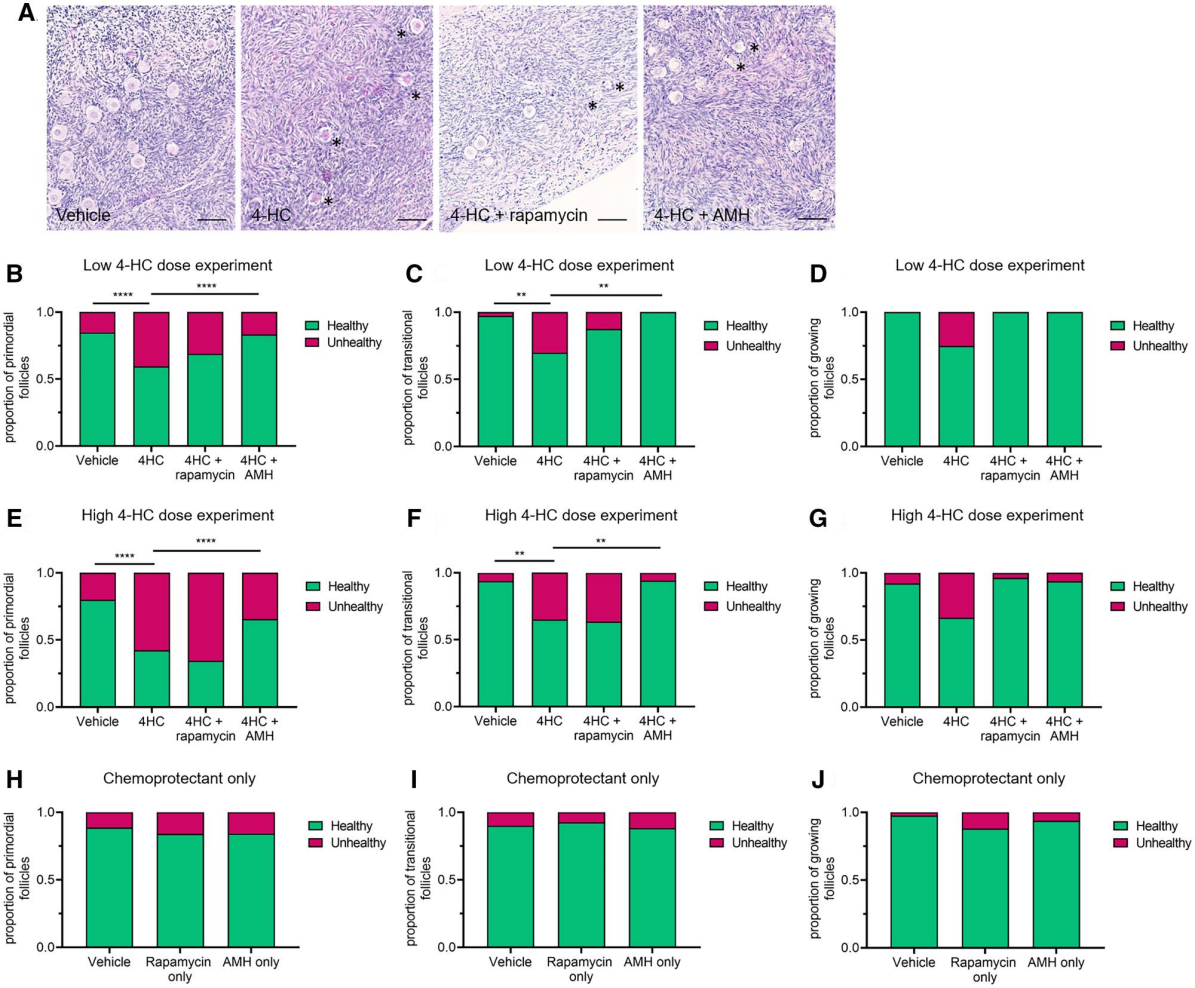
**Primordial, transitional, and growing follicle health in human ovarian cortex following 24 h exposure to 4-HC.** (**A**) Representative histological images of cultured ovarian cortical tissue belonging to each of the four experimental groups, with unhealthy follicles identified by (*). Scale bar = 100 µm. Proportion of primordial, transitional, and growing follicles classified as healthy or unhealthy in experiments with low 0.2 µM doses of 4-hydroperoxycyclophosphamide (4-HC) (**B–D**), high 2 µM doses of 4-HC (**E, F**), and chemoprotectants (anti-Mullerian hormone (AMH) and rapamycin) only (**H–J**). N = 3–7, 280 primordial follicles, 128 transitional follicles, 28 growing follicles for low-dose experiments (105 follicles in vehicle group, 174 follicles in 4-HC group, 64 follicles in 4-HC+rapamycin group, and 93 follicles in 4-HC+AMH group). N = 4–6, 1074 primordial follicles, 150 transitional follicles, 61 growing follicles for high-dose experiments (306 follicles in vehicle group, 172 follicles in 4-HC group, 528 follicles in 4-HC+rapamycin group, and 279 follicles in 4-HC+AMH group). N = 4, 1825 primordial follicles, 314 transitional follicles, and 171 growing follicles for chemoprotectant only experiments (672 in vehicle group, 752 in AMH only group, and 886 in rapamycin only group). ****P < 0.0001, **P < 0.01; Fisher’s exact test.

In these experiments, the majority of follicles (both non-growing and growing) were classed as healthy based on the appearance of the oocyte, with unhealthy follicles having oocytes that had eosin-stained nuclei, shrunken cytoplasm, or overall distorted morphology; there was generally no evidence of unhealthy pyknotic granulosa cells (Fig. 2A). The proportion of unhealthy non-growing follicles across all vehicle cultures was 12.9 ± 2.8%. Culture with either dose of 4-HC caused an increase in the proportion of unhealthy primordial (*P* < 0.0001, both doses) and transitional follicles (*P* < 0.01 for low dose and *P* < 0.01 for high dose) compared to the vehicle (Fig. 2B and C (low dose) and Fig. 2E and F (high dose)). The data suggest a dose–response effect of 4-HC, with the proportion of unhealthy primordial follicles higher in the high-dose cultures (57.7 ± 13.8%) compared to 40.5 ± 5.9% in the low-dose cultures (Fig. 2B and E), however, no statistical comparisons were made since these were separate experiments. While the addition of rapamycin did not significantly reduce the proportion of unhealthy primordial or transitional follicles at either dose of 4-HC, the presence of AMH significantly attenuated follicle damage, with the combined proportion of unhealthy primordial and transitional follicles in these groups being approximately half, or less than half, that observed in the 4-HC alone group at both doses (*P* < 0.0001) (Fig. 2B, C, E, and F). Furthermore, in the low-dose experiments, there was no significant difference in the proportion of unhealthy primordial follicles or unhealthy transitional follicles between the 4-HC+AMH treated groups and vehicle (15.1 ± 6.9% unhealthy primordial follicles in vehicle vs 16.7 ± 16.7% in 4-HC+AMH group and 2.8 ± 2.8% unhealthy transitional follicles in vehicle vs 0 ± 0% in 4-HC+AMH group). In a series of separate experiments, we assessed the effect of rapamycin or AMH on follicle health and showed that there was no significant difference in the proportion of unhealthy primordial, transitional, or growing follicles compared to the vehicle control (Fig. 2H, I, and J). These data therefore show that AMH but not rapamycin can protect non-growing follicles from the damage caused by the effects of 4-HC during culture.

The proportion of growing (i.e. primary and secondary) follicles was low, as expected given the short duration of the *in vitro* culture. While the data appear to demonstrate similar toxicity of 4-HC on growing follicles as on non-growing follicles (Fig. 2D and G) and suggest that both rapamycin and AMH may have chemoprotective effects on growing follicles (Fig. 2D and G), the results were not significant.

### 4-HC exposure induces PI3K signalling upregulation in human ovarian cortex *in vitro*, which is inhibited by culture with rapamycin and AMH

**Figure 3.**
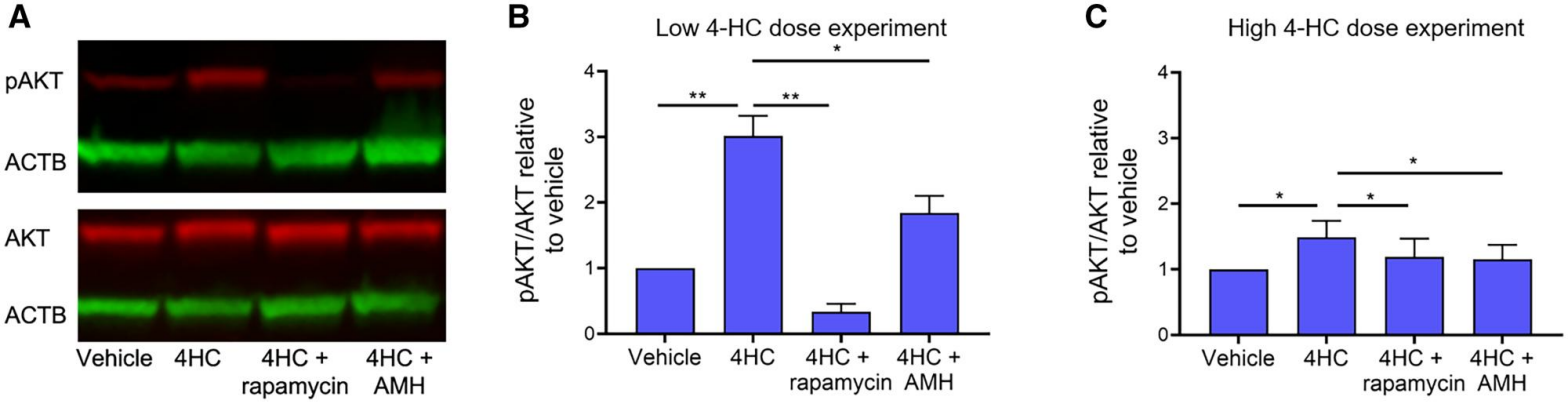
**pAKT/AKT expression in human ovarian cortex following 24 h exposure to 4-HC.** (**A**) Representative Western blot depicting expression of pAKT (red band at ∼60 kDa), AKT (red band at ∼60 kDa), and ACTB (green band at ∼40–45 kDa) in human ovarian cortex across the four experimental groups. Note pAKT and AKT expression were measured in the same ovarian cortical sample. (**B, C**) Quantification of pAKT expression relative to AKT expression for experiments with low 0.2 µM doses of 4-hydroperoxycyclophosphamide (4-HC) (B) and high 2 µM doses of 4-HC (C), with or without the chemoprotectants (anti-Mullerian hormone (AMH) and rapamycin), relative to vehicle. N = 4–6 ovarian biopsies. *P < 0.05, **P < 0.01; Mann–Whitney test.

Human ovarian cortical strips were cultured with 0.2 or 2 µM 4-HC for 24 h and Western blotting was used to quantify the phosphorylation of the PI3K signalling mediator AKT (Fig. 3A). pAKT and AKT expressions were normalized to the reference protein ACTB, and normalized quantities were plotted relative to vehicle-cultured cortical strips. Culture with low-dose (0.2 µM) 4-HC caused an approximate 3-fold increase in AKT phosphorylation (*P* < 0.01), while high-dose (2 µM) 4-HC exposure resulted in a 1.5-fold increase in pAKT expression (*P* < 0.05) (Fig. 3B and C). Increased phosphorylation of AKT is consistent with upregulation of PI3K signalling (Hemmings and Restuccia, 2012), suggesting that the presence of 4-HC can activate this signalling pathway in the human ovarian cortex. Concurrent with this, experiments were undertaken to investigate the ability of rapamycin or AMH to attenuate the effects of 4-HC on PI3K signalling by adding these chemicals to the culture alongside 4-HC itself. In the low-dose 4-HC experiments, culture with rapamycin or AMH attenuated the 4-HC-induced AKT phosphorylation by approximately 10- and 1.5-fold, respectively (Fig. 3B) (*P* < 0.01 for rapamycin, *P* < 0.05 for AMH vs 4HC alone). A similar inhibitory effect was observed in the high-dose 4-HC culture experiments, with both rapamycin and AMH (both *P* < 0.05) (Fig. 3C). Rapamycin and AMH showed similar protective efficacy against this higher dose of 4-HC, although the magnitude of all changes was less than in the low-dose experiments.

**Figure 4.**
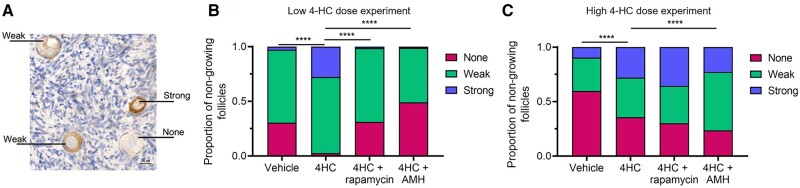
**pFOXO3A staining intensity in human ovarian cortex following 24 h exposure to 4-HC.** (**A**) Representative image of pFOXO3A immunostaining in human ovarian cortical tissue identifying primordial follicle oocytes with no, weak, or strong pFOXO3A staining determined using FIJI. (**B, C**) Quantification of the proportion of primordial and transitional follicles with no, weak, or strong pFOXO3A staining in experiments with low 0.2 µM doses of 4-hydroperoxycyclophosphamide (4-HC), (B) and high 2 µM doses of 4-HC (C), with or without the chemoprotectants (anti-Mullerian hormone (AMH) and rapamycin). N = 3–7, 408 non-growing follicles counted for low-dose experiments. N = 4–6, 1074 non-growing follicles counted for high-dose experiments. ****P < 0.0001; Fisher’s exact test.

To complement the Western blotting data, immunostaining for phosphorylated FOXO3A (pFOXO3A) was carried out to investigate the PI3K signalling pathway in primordial follicles specifically. FOXO3A is an effector of PI3K signalling in oocytes, and upon activation by phosphorylation and nuclear export, it triggers primordial follicle activation and growth (John *et al.*, 2008). The nuclear-cytoplasmic translocation of FOXO3A has been used to measure PI3K signalling activation; however, as this was found to be unreliable in our human ovarian cortical tissue, quantification of pFOXO3A immunostaining intensity was used instead. Following a 24-h exposure to low 0.2 µM or high 2 µM dose 4-HC, oocytes within non-growing follicles (defined as primordial and transitional follicles) were classified as either having no, weak, or strong pFOXO3 staining, determined objectively by FIJI and image mean grey values (Fig. 4A). In the low-dose 4-HC group, there was a higher proportion of strongly stained pFOXO3A oocytes compared to the vehicle group (27.7 ± 8.4% vs 2.7 ± 2.7%, *P* < 0.0001), consistent with upregulation of PI3K signalling. Furthermore, addition of either rapamycin or AMH during culture was able to reduce this 4-HC-induced effect on pFOXO3A expression, with the proportion of strongly-stained oocytes in these groups being very similar to that observed in the vehicle (1.4 ± 1.4% in rapamycin+4-HC cultures and 1.2 ± 1.2% in AMH+4-HC cultures, both *P* < 0.0001 vs 4-HC alone) (Fig. 4B). These data support the Western blotting findings and suggest that low 0.2 µM dose 4-HC can upregulate PI3K signalling in the human ovarian cortex, and this can be attenuated by culture with either rapamycin or AMH.

In the high-dose cultures, there was also an increase in the proportion of strongly stained pFOXO3A oocytes compared to the vehicle group (28.1 ± 9.9% vs 9.6 ± 6.2%, *P* < 0.0001) (Fig. 4C). Addition of rapamycin had no significant effect, but AMH resulted in a reduction in the proportion of non-growing follicles to 22.8 ± 8.2% strongly stained pFOXO3A oocytes (*P* < 0.0001 vs 4-HC alone), a small but significant decrease compared to that observed in the 4-HC alone group. Although the experimental design prevents us from definitively concluding this, the data suggest there is no difference in the proportion of strongly stained pFOXO3A oocytes between the low and high 4-HC dose, and the pAKT Western blot data (Fig. 3) showed a similarly unexpected dose–response trend. These data thus indicate that the effects of 4-HC on PI3K signalling in the human ovarian cortex do not follow a typical dose–response relationship, yet our data consistently show the ability of AMH to inhibit these effects.

## Discussion

It is well-recognized that chemotherapeutic agents affect the growing ovarian follicles and the oocytes they contain (Spears *et al.*, 2019). However, it is the risk to the non-growing reserve of primordial follicles underpinning life-long ovarian function that is the most important in determining long-term consequences for female reproductive function. There are two ways by which chemotherapy is thought to cause primordial follicle loss: direct toxicity to primordial follicles with subsequent death and/or indirect depletion of primordial follicles through over-recruitment. The latter is thought to occur as a consequence of the loss of negative feedback normally provided by AMH from the growing follicles, which are highly susceptible to chemotherapeutic agents targeting proliferative cells, or by direct effects on the primordial follicles to induce increased rates of growth initiation. Data supporting these mechanisms have been largely obtained using *in vitro* and *in vivo* rodent models; however, the outcomes of much of this work are conflicting, with few publications reporting evidence of both primordial follicle damage and premature follicle activation (Bellusci *et al.*, 2019; Kashi *et al.*, 2023). In this study, we have shown for the first time, that AMH protects primordial and transitional follicle health within the human ovary from the effects of exposure to an active metabolite of cyclophosphamide, 4-HC. The 4-HC also upregulates PI3K signalling (a primordial follicle activation pathway), and we have shown that that pathway is also attenuated by AMH. The mTOR inhibitor rapamycin was able to prevent the upregulation of the PI3K pathway, and indeed was more effective in that regard than AMH, but did not mitigate the adverse effects of 4-HC on follicle health. Ultimately, these data suggest that both AMH and rapamycin may have protective effects on primordial follicles from the harmful side-effects of chemotherapy treatment, and thereby preserve a woman’s fertility.

Apoptosis of oocytes and granulosa cells is a key mechanism thought to underlie primordial follicle loss following chemotherapy treatment in general and cyclophosphamide exposure in particular (Stringer *et al.*, 2023). In this study, we observed a clear effect on primordial and transitional follicle health, with the proportion of unhealthy primordial follicles increasing by a third between low- and high-dose 4-HC exposure. Follicle health was determined histologically, with follicles deemed unhealthy by well-established markers of impaired follicle health (McLaughlin and Telfer, 2010; McLaughlin *et al.*, 2014), i.e. oocyte-specific nuclear condensation or shrunken cytoplasm, or an overall distorted follicle morphology. In support of these findings, cyclophosphamide treatment was shown histologically to impair the survival and development of ovarian follicles derived from tissue biopsies taken from female cancer patients after the initiation of chemotherapy (Asadi Azarbaijani *et al.*, 2015). Furthermore, cyclophosphamide caused a peak reduction in primordial follicle density at 48-h post-treatment in a human foetal ovarian xenograft model (Oktem and Oktay, 2007a), although the non-uniformity of primordial follicle distribution in these xenografts should be considered when interpreting these results. These authors however did provide evidence of apoptosis evaluated via TUNEL assay, showing that damage to primordial follicles took place as early as 12 h after the injection of cyclophosphamide, and in later work, they used single-cell RNA sequencing on laser-captured individual primordial follicle oocytes from a similar model to show a significant decrease in expression of several anti-apoptotic genes (Titus *et al.*, 2021). TUNEL assays in combination with immunostaining for DNA damage and apoptotic cascade markers including cleaved-CASP3, γH2AX, and TAP63 have generally been used to confirm chemotherapy-induced damage to primordial follicles in many rodent studies, with such endpoints investigated between 2 and 24 h following the insult (Gonfloni *et al.*, 2009; Soleimani *et al.*, 2011; Kerr *et al.*, 2012; Luan *et al.*, 2019; Nguyen *et al.*, 2019; Lopes *et al.*, 2020; Stringer *et al.*, 2020). These methods have also been used in a recent study investigating *in vivo* chemotherapy damage in pre- and post-pubertal human ovarian tissue (Devos *et al.*, 2023). However, in that study, it is unclear how long after the chemotherapy treatment these analyses were conducted; this could be relevant, since the specificity of the window of effect may potentially explain why not all studies report findings of apoptosis or direct follicle damage following chemotherapy exposure. Nevertheless, while our study demonstrates that 4-HC treatment affected primordial and transitional follicle health, we did not explore whether this triggered an apoptotic cascade or follicle death by non-classical pathways, and more importantly, whether the tissue is able to repair/recover from this damage; for human studies, this would require a culture or xenograft model that supports the long-term growth of primordial follicle oocytes (Oktem and Oktay, 2007a; McLaughlin *et al.*, 2018).

The PI3K/AKT pathway is one of the major signalling pathways that coordinates the activation, differentiation and growth of primordial follicles through relaying extracellular signals from growth factors and cytokines in the ovary (Maidarti *et al.*, 2019). Upregulation of PI3K signalling within the oocyte triggers a cascade of kinase reactions that converge on the transcription factor FOXO3A, whose phosphorylation results in its export from the nucleus to the cytoplasm, resulting in follicle activation (John *et al.*, 2008). Using whole tissue Western blotting, we observed an upregulation in AKT phosphorylation in 4-HC cultured tissue, indicative of PI3K signalling activation. This effect was observed at both 4-HC doses investigated, however the higher 4-HC dose elicited significantly less AKT phosphorylation than the lower dose; it is possible that the effect on AKT phosphorylation is specific to lower doses of 4-HC, with higher doses predominantly affecting ovarian follicle and stromal cell health. However, direct comparison of dose and effect relationships with other studies is potentially difficult, with many studies typically adopting an *in vivo* cyclophosphamide exposure model, making it unclear exactly what individual metabolite doses the ovary received, and those that have used a metabolite-specific *in vitro* approach did not quantify PI3K signalling (Kano *et al.*, 2017; Luan *et al.*, 2019). Reports detailing evidence of premature primordial follicle growth following cyclophosphamide exposure have either used whole tissue Western blotting for downstream mediators of PI3K signalling (e.g. pAKT pMTOR, p70-S6K) and/or collected data reflecting changes in follicle proportions to suggest follicle growth activation (Kalich-Philosoph *et al.*, 2013; Sonigo *et al.*, 2019). The PI3K signalling pathway is ubiquitous in many cell types, thus data from whole tissue Western blotting may include PI3K signalling activation in granulosa and stromal cells as well as in oocytes. Furthermore, activation of PI3K signalling is also involved in a diverse set of biological processes including cell growth, migration, and survival (Carracedo and Pandolfi, 2008; Georgescu, 2010), autophagy (Rabanal-Ruiz *et al.*, 2017), stress response (Bakkenist and Kastan, 2004), and its co-expression with c-PARP in mouse oocytes suggests that this pathway can also mediate oocyte apoptosis (Luan *et al.*, 2019). Therefore, such Western blotting data should be subsequently visualized *in situ* with immunolabelling of pFOXO3A, a well-established indicator of primordial follicle activation; this may be better than Western blotting as pFOXO3A expression has also been observed in granulosa cells and its effect on follicle growth is unclear (Matsuda *et al.*, 2011; Mikaeili *et al.*, 2016). Our study stratified and quantified pFOXO3A expression, showing a significant increase in expression specifically within oocytes, and this combined with our Western blotting data, suggests that the PI3K pathway is upregulated in non-growing follicles following 24 h culture with 4-HC. We did not however translate this finding into differences in growing follicles, therefore cannot definitively conclude that 4-HC causes primordial follicle loss through premature follicle depletion. Such data are complicated to collect and interpret in human ovarian cortical culture studies, since primordial follicles are not evenly distributed through the cortex and the biopsies obtained, and tissue samples cultured are not necessarily reflective of the total ovary follicle composition. Follicle data are therefore often reported as proportions of total follicle numbers. However, this can be misleading as it is difficult to ascertain whether changes observed in specific follicle stages are a result of loss of one follicle stage (e.g. by apoptosis), or reflect true growth of a follicle from one stage to the next. Thus, studies using proportional follicle staging data to support primordial follicle activation in the human ovary following chemotherapy treatment should be interpreted with caution (Lande *et al.*, 2017). This caveat is easily circumnavigated in mouse studies where whole ovaries can be obtained, allowing changes in total follicle number to be reported alongside follicle proportions to enable a more robust interpretation of data.

Improvements in our understanding of the direct and indirect effects of chemotherapy treatment on the ovary will support the development of knowledge-based approaches towards prevention of adverse effects. Significant strides have been made in this field of chemoprotection using rodent models, however, human data are scarce (Spears *et al.*, 2019). Here we report for the first time, the efficacy of AMH to attenuate primordial follicle damage caused by 4-HC exposure, with both AMH and rapamycin demonstrating the ability to reduce 4-HC-induced PI3K signalling activation in the human ovary. The chemoprotective effects of AMH were first shown by Kano *et al.* (2017), who demonstrated that AMH administration (via AAV9 gene therapy or pump delivery of recombinant human AMH) could reversibly block primordial follicle activation in mice and reduce the effects of carboplatin, doxorubicin, or cyclophosphamide on follicle loss in a mouse tumour model. AMH also proved to be an effective chemoprotectant in two separate cyclophosphamide-treated prepubertal mouse models (Roness *et al.*, 2019; Sonigo *et al.*, 2019) and based on their data, the authors hypothesized that AMH may regulate autophagy to protect primordial follicles and limit follicle depletion induced by cyclophosphamide (Sonigo *et al.*, 2019). These studies actually focused on the effect of AMH on preventing primordial follicle loss through upregulated PI3K signalling and premature follicle activation, however, our data also demonstrate a role for AMH in protecting primordial follicles from direct chemotherapy-induced damage. This has previously been explored with successful results using inhibitors targeting components of the apoptotic pathway (Gonfloni *et al.*, 2009; Li *et al.*, 2014; Meng *et al.*, 2014; Nguyen *et al.*, 2018), however, the delivery of such inhibitors may prove problematic with regards to potential interference with the effects of chemotherapy on the tumour itself. Nevertheless, AMH treatment was shown not to interfere with the cytotoxic actions of cyclophosphamide on breast cancer cells or an *in vivo* model of human leukaemia (Roness *et al.*, 2019) and AMH has been shown not to affect tumour growth (Kano *et al.*, 2017). This combined with its ovary-specific functions, makes it a promising candidate for chemoprotection in the ovary. Although autophagy has been proposed (Sonigo *et al.*, 2019), the mechanism underlying the chemoprotective capacity of AMH remains elusive. scRNA sequencing of neonatal mouse ovaries exposed to supraphysiological doses of AMH showed that AMH had a modest effect on the oocyte transcriptome, in contrast to other cell types including granulosa, ovarian surface epithelium, and stromal cells, where a significant number of differentially expressed genes were observed (Meinsohn *et al.*, 2021). AMH receptor 2 expression has also been reported in these cell types but not in oocytes (Weenen *et al.*, 2004; Meinsohn *et al.*, 2021), suggesting the chemoprotective effect of AMH on non-growing follicle oocytes may be indirect, and could potentially involve maintaining or stabilizing the oocyte–somatic cell interactions. Furthermore, the effect of AMH is likely to be dose-dependent, with a separate transcriptomic study showing an AMH dose-related and time-dependent response (Ur Rehman *et al.*, 2018). Nevertheless, our knowledge is lacking on the effects of AMH on cell-to-cell communication and extracellular matrix composition, both important variables that can dictate the physiological state of ovaries (Fraire-Zamora *et al.*, 2023). Answers to these questions will no doubt shed light upon the value of AMH for chemoprotection.

Our data demonstrating the ability of rapamycin to attenuate 4-HC-induced upregulated PI3K signalling in the human ovary is perhaps expected, given the mechanism of action of this well-known inhibitor; this effect has also been reported in multiple rodent studies alongside the chemoprotective efficacy of mTOR inhibitors in general (Goldman *et al.*, 2017; Zhou *et al.*, 2017; Tanaka *et al.*, 2018; Chen *et al.*, 2022). Although not significant in our data, possibly due to limited numbers of growing follicles, other work in rodents has demonstrated the effectiveness of rapamycin treatment in attenuating cyclophosphamide-induced growing follicle damage (Zhou *et al.*, 2017; Chen *et al.*, 2022). Although human ovary cortex cultured with rapamycin for 6 days had a high proportion of follicles with fragmented or shrunken oocytes (McLaughlin *et al.*, 2011), this adverse effect on oocyte health was not observed in rodent studies or human ovarian cortical xenotransplantation experiments (Yorino and Kawamura, 2020) nor are there any reports describing adverse reproductive outcomes following rapamycin treatment. Lastly, regarding the study of chemoprotectants, recent evidence suggests there may be significant value in investigating protective agents in combination, with data showing that co-treatment of temsirolimus and AMH completely prevented cyclophosphamide-induced primordial follicle loss (Kashi *et al.*, 2023).

In conclusion, this work is among the first human data to demonstrate an effect of cyclophosphamide on both direct primordial follicle damage and PI3K signalling, and potentially primordial follicle activation. Furthermore, we have provided novel evidence supporting two different mechanisms for the chemoprotective efficacy of AMH in the human ovary; while rapamycin also prevented PI3K signalling, its effects on follicle protection were unclear. These data therefore suggest that administration of AMH alongside chemotherapy treatment may have significant benefit in protecting the ovarian reserve, and in fact there may be potential for benefit from both AMH and rapamycin as suggested by other rodent studies (Kashi *et al.*, 2023). Important future work in this field will require consideration of the sustained effects of chemotherapy treatment and potential protectants to ensure that these agents do not impair the developmental competence of oocytes or lead to the survival of oocytes with accumulated DNA damage, which could have adverse consequences for the potential offspring.

## Supplementary Material

dead255_Supplementary_Figure_S1

dead255_Supplementary_Table_S1

## Data Availability

The data underpinning this article will be shared upon reasonable request to the corresponding author.
