# Correction to: Anti-Mullerian hormone attenuates both cyclophosphamide-induced damage and PI3K signalling activation, while rapamycin attenuates only PI3K signalling activation, in human ovarian cortex *in vitro*

**DOI:** 10.1093/humrep/deae034

**Published:** 2024-02-16

This is a correction to: Roseanne Rosario, Hazel L Stewart, Norah Spears, Evelyn E Telfer, Richard A Anderson, Anti-Mullerian hormone attenuates both cyclophosphamide-induced damage and PI3K signalling activation, while rapamycin attenuates only PI3K signalling activation, in human ovarian cortex *in vitro*, Human Reproduction, Volume 39, Issue 2, February 2024, Pages 382–392, https://doi.org/10.1093/humrep/dead255

The authors of the above article would like to apologise for a typographical error in the concentration of anti-Müllerian hormone (AMH) listed in the ‘Ovarian cortical tissue collection, preparation, and culture’ section of the Materials and methods.

The AMH concentration is mistakenly stated as:

“*200ng/µl recombinant human AMH*”

Instead of

“*200ng/ml recombinant human AMH*”

The section of Materials and methods should read as:

At least two to three cortical fragments were assigned to one of four treatment groups: vehicle (DMSO), 4-HC alone (Niomech IIT GmbH, Germany, diluted in DMSO), 4-HC + 100nM rapamycin (LC Laboratories, USA, prepared according to manufacturer’s instructions), or 4-HC + 200ng/ml recombinant human AMH (R&D Systems, UK, prepared according to manufacturer’s instructions).

The authors would like to assure readers that this typographical error does not affect any results, conclusions or other content of the article.

The electronic version of this article has been updated at https://doi.org/10.1093/humrep/dead255.

