# Supplementary material for: Anti-Mullerian hormone attenuates both cyclophosphamide-induced damage and PI3K signalling activation, while rapamycin attenuates only PI3K signalling activation, in human ovarian cortex in vitro
Source: Hum Reprod. 2023 Dec 9;39(2):382–92. doi: 10.1093/humrep/dead255 (PMC10833070; doi:10.1093/humrep/dead255)
Supplement: dead255_Supplementary_Figure_S1 [file dead255_supplementary_figure_s1.pdf]

human
reproduction

Human Reproduction, 2023, 1–1
https://doi.org/10.1093/humrep/dead255
Supplementary Data

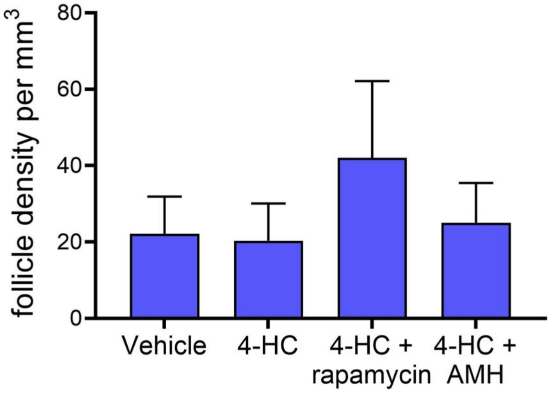

Supplementary Figure S1. Follicle density in ovarian samples used for the high-dose experiments. Quantification of ovarian follicle density in biopsies used for experiments with high doses of 4-hydroperoxycyclophosphamide (4-HC) with or without the chemoprotectants (anti-Mullerian hormone (AMH) and rapamycin). N = 4–6.
