# Supplementary material for: Anti-Mullerian hormone attenuates both cyclophosphamide-induced damage and PI3K signalling activation, while rapamycin attenuates only PI3K signalling activation, in human ovarian cortex in vitro
Source: Hum Reprod. 2023 Dec 9;39(2):382–92. doi: 10.1093/humrep/dead255 (PMC10833070; doi:10.1093/humrep/dead255)
Supplement: dead255_Supplementary_Table_S1 [file dead255_supplementary_table_s1.pdf]

OXFORD

human reproduction

Human Reproduction, 2023, 1–1
https://doi.org/10.1093/humrep/dead255
Supplementary Data

Supplementary Table S1. Patient age and total follicle number counted for experiments with low doses or high doses of 4-hydroperoxycyclophosphamide (4-HC) or chemoprotectants only (anti-Mullerian hormone (AMH) and rapamycin).

Experiment	Patient ID	Age	Total follicles counted
Low-dose 4-HC experiments	OVO920	37	10
	OVO921	29	110
	OVO925	32	35
	OVO963	31	33
	OVO968	41	174
	OVO970	27	50
	OVO976	33	24
High-dose 4-HC experiments	OVO1017	33	31
	OVO1018	34	19
	OVO1036	25	848
	OVO1048	32	72
	OVO1069	30	237
	OVO1055	21	78
Rapamycin and AMH only experiments	OVO1145	38	215
	OVO1146	33	1565
	OVO1147	36	354
	OVO1148	28	176
